# Discrepant Biopsy and Serological Findings in Nephrotic Syndrome: A Case of Phospholipase A2 Receptor (PLA2R)-Positive Membranous Nephropathy Mimicking Post-infectious Glomerulonephritis

**DOI:** 10.7759/cureus.97302

**Published:** 2025-11-20

**Authors:** Sally Hamad, Hussein Ali Kadhim Al-Iedani, Gagan Suresha, Noor Alwan, AbdulRahman Al-Mohammed

**Affiliations:** 1 General Medicine, Peterborough City Hospital, Peterborough, GBR; 2 General Medicine, Basrah Medical College, Basrah, IRQ; 3 General Medicine, Novosibirsk State University, Novosibirsk, RUS

**Keywords:** anti-phospholipase a2 receptor antibodies, biopsy, membranous nephropathy, microscopic colitis, nephropathy, nephrotic syndrome, pla2r, primary membranous nephropathy, renal biopsy, rituximab

## Abstract

Anti-phospholipase A2 receptor (PLA2R) antibody testing has transformed the diagnosis of membranous nephropathy in adults with nephrotic syndrome. However, discrepancies between serological and histopathological findings can create significant diagnostic and therapeutic uncertainty. We report the case of a 72-year-old woman with heavy proteinuria, hypoalbuminaemia, and markedly elevated PLA2R antibodies, whose initial renal biopsy suggested a resolving post-infectious glomerulonephritis. Owing to persistent nephrotic syndrome, a second biopsy performed four weeks later revealed membranous nephropathy, prompting treatment with prednisolone and rituximab. Her course was complicated by steroid-refractory microscopic colitis, rapidly progressive vitiligo, severe fluid overload resistant to high-dose diuretics, and recurrent nocturnal desaturation, revealing previously undiagnosed chronic obstructive pulmonary disease.

This case highlights the importance of repeat renal biopsy when serological and histological findings diverge and illustrates the challenges of delivering immunosuppression in patients with multiple comorbidities. Early multidisciplinary input was essential in establishing the diagnosis and planning treatment.

## Introduction

Membranous nephropathy is the leading cause of nephrotic syndrome in adults and is characterised by subepithelial immune complex deposition along the glomerular basement membrane [1]. The identification of circulating anti-phospholipase A2 receptor (PLA2R) antibodies has revolutionised diagnosis by providing a highly specific and sensitive marker for primary disease [2]. Nevertheless, renal biopsy remains the diagnostic gold standard, particularly when clinical or serological findings are atypical [3].

Discordance between PLA2R serology and histopathology, though uncommon, presents a significant clinical challenge [4]. In such cases, clinicians must weigh the risks of immunosuppression against the possibility of an alternative diagnosis, and a repeat biopsy may be required to clarify disease evolution or mixed pathology [5-7].

Therapeutic decision-making becomes even more complex in the presence of comorbid conditions such as chronic gastrointestinal inflammation or dermatological autoimmunity, which may both confound diagnosis and influence the safety of immunosuppressive therapy [5,8]. Multidisciplinary input is therefore critical in managing these patients [9].

We report the case of a 72-year-old woman with PLA2R-positive nephrotic syndrome, whose first renal biopsy suggested a resolving post-infectious glomerulonephritis, while a second biopsy, performed four weeks later, confirmed membranous nephropathy. Her management was further complicated by steroid-refractory microscopic colitis, progressive vitiligo, severe diuretic-resistant fluid overload, and undiagnosed chronic obstructive pulmonary disease (COPD). This case highlights the diagnostic uncertainty created by discordant serology and histology, and underscores the value of repeat biopsy and coordinated multidisciplinary care.

## Case presentation

A 72-year-old woman with a history of deep vein thrombosis, steroid-refractory microscopic colitis, and long-term cigarette smoking was admitted in July 2025 with progressive bilateral lower limb and sacral oedema, weight gain, and new-onset breathlessness. She reported unintentional weight loss of approximately three stone over the preceding two years. Medications at admission included furosemide, ramipril, apixaban, and loperamide. Two weeks before presentation, she had received a short course of amoxicillin for a urinary tract infection. There was no history of recent travel, nephrotoxic drug exposure, or systemic symptoms.

On examination, she was afebrile, normotensive, and saturating 93% on room air. She had pitting oedema to the mid-thigh and mild sacral oedema. The abdomen was distended but non-tender. Cardiorespiratory and neurological examinations were otherwise unremarkable.

Initial investigations (Table 1) revealed a rising serum creatinine of 212 µmol/L (estimated glomerular filtration rate 20 mL/min/1.73 m²), serum albumin 20 g/L, and a urine albumin level exceeding 4000 mg/L, consistent with nephrotic-range proteinuria. Antinuclear antibody was weakly positive with anti-Ro reactivity; anti-neutrophil cytoplasmic antibodies were negative; complement levels were normal; and serum immunoglobulins were polyclonal. Anti-PLA2R antibodies were markedly elevated at 686 RU/mL. Tumour markers, including alpha-fetoprotein (AFP), carcinoembryonic antigen (CEA), carbohydrate antigen 19-9 (CA19-9), and cancer antigen 125 (CA-125), were within normal ranges.

**Table 1:** Initial Laboratory Investigations Elevated serum creatinine and a reduced estimated glomerular filtration rate (eGFR) indicate significant renal impairment. The markedly low serum albumin level, combined with an extremely high urine albumin concentration, confirms nephrotic-range proteinuria and supports the diagnosis of nephrotic syndrome. Positive antinuclear antibody (ANA) and anti-Ro (Sjögren’s Syndrome Antigen A, or SSA) antibody results suggest an underlying autoimmune process. Negative anti-neutrophil cytoplasmic antibodies (ANCA) and normal complement levels (C3 and C4) help exclude vasculitis. Most notably, the patient's anti-phospholipase A2 receptor (PLA2R) antibody level is profoundly elevated, strongly supporting a diagnosis of primary membranous nephropathy, despite initial biopsy findings suggesting post-infectious glomerulonephritis.

Parameter	Result	Normal Reference Range
Serum Creatinine	212.0 (µmol/L)	45.0 - 84.0 (µmol/L)
Estimated Glomerular Filtration Rate (eGFR)	20.0 (mL/min/1.73 m²)	≥ 60.0 (mL/min/1.73 m²)
Serum Albumin	20.0 (g/L)	35.0 - 50.0 (g/L)
Urine Albumin	> 4000.0 (mg/L)	< 30.0 (mg/L)
Urine Creatinine	4.50 (mmol/L)	6 - 13 (mmol/L)
Antinuclear Antibody (ANA)	Positive	Negative
Anti-Ro (Sjögren’s Syndrome Antigen A, or SSA) Antibody	Positive	Negative
Anti-neutrophil Cytoplasmic Antibody (ANCA)	Negative	Negative
C3	1.01 (g/L)	0.75 - 1.65 (g/L)
C4	0.15 (g/L)	0.14 - 0.54 (g/L)
Immunoglobulin G	12.1 (g/L)	6.0 - 16.0 (g/L)
Immunoglobulin A	2.67 (g/L)	0.8 - 4.0 (g/L)
Immunoglobulin M	4.17 (g/L)	0.4 - 2.5 (g/L)
Anti-phospholipase A2 Receptor (PLA2R) Antibody	686.0 (RU/mL)	< 14.0 (RU/mL)

**Figure 1:**
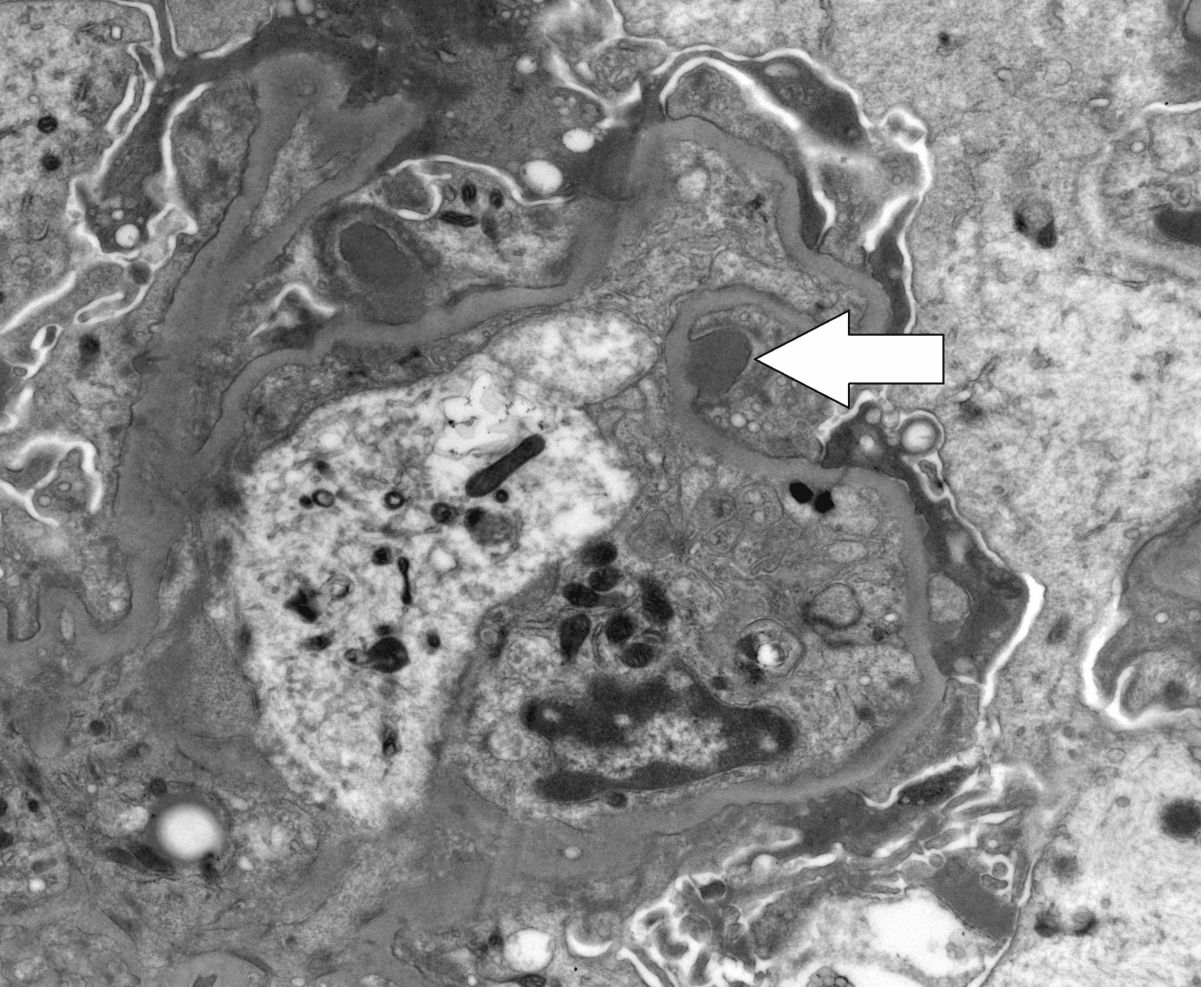
Electron Microscopy of Initial Renal Biopsy Demonstrating Subepithelial “Hump-Like” Deposits Electron micrograph (×2500) from the first renal biopsy, showing irregular subepithelial electron-dense “hump-like” deposits (white arrow) along the glomerular basement membrane, with partial foot process effacement. These features are consistent with a resolving post-infectious glomerulonephritis pattern rather than membranous nephropathy.

A first renal biopsy, performed in July 2025, demonstrated widespread foot process effacement with subepithelial “hump” deposits and IgG/C3 positivity, consistent with a resolving post-infectious glomerulonephritis rather than membranous nephropathy (Figure 1).

Because of persistent nephrotic syndrome and diagnostic uncertainty, a second renal biopsy was performed several weeks after the first biopsy. This showed a histological picture consistent with membranous nephropathy, clarifying the diagnosis.

**Figure 2:**
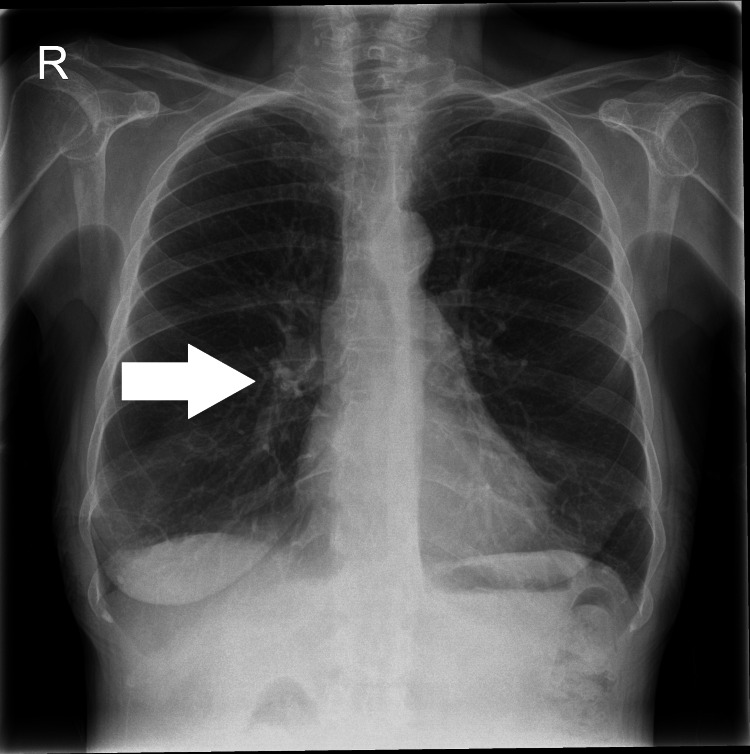
Chest Radiograph Highlighting Bronchovascular Markings Posteroanterior chest radiograph demonstrating prominent bronchovascular markings (white arrow), consistent with chronic airway changes.

Imaging during admission supported a multisystem picture. Chest radiography demonstrated hyperinflated lungs, with coarse bronchovascular markings (Figure 2).

**Figure 3:**
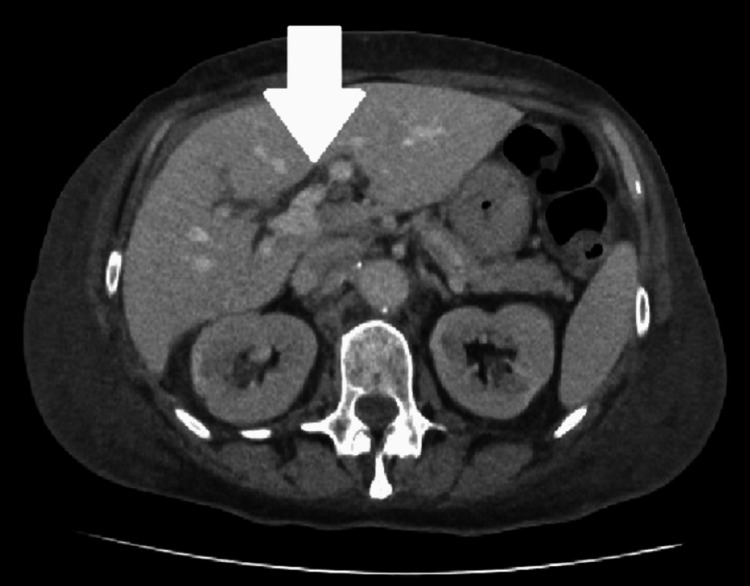
Contrast-Enhanced CT Abdomen Showing Periportal Oedema Axial CT image of the abdomen demonstrating periportal oedema (white arrow), with surrounding anasarca. Small-volume ascites and soft tissue oedema are also present, consistent with fluid overload secondary to nephrotic syndrome.

Echocardiography revealed preserved left ventricular systolic function, with impaired diastolic relaxation and borderline left atrial dilatation. Computed tomography of the abdomen and pelvis showed marked anasarca, periportal oedema, small pleural effusions, and no evidence of malignancy (Figure 3).

Despite high-dose intravenous furosemide (up to 320 mg/24 h), combined with metolazone, albumin infusions, fluid restriction, and dietary modification, the diuretic response was limited. Gastroenterology advised conservative management of her microscopic colitis, and dermatology confirmed progressive vitiligo but recommended avoiding additional systemic immunosuppression beyond that required for her renal disease.

From mid-admission, she developed recurrent nocturnal desaturation (oxygen saturation 88%-91%) with wheeze; arterial blood gases showed chronic hypercapnia. She was treated empirically with nebulised bronchodilators and assessed for previously undiagnosed COPD.

Following the second biopsy, she was commenced on oral prednisolone and discharged on a weaning regimen. Rituximab therapy was initiated as two infusions, administered over a two-week interval.

A multidisciplinary approach, involving renal, gastroenterology, dermatology, respiratory, haematology, and gynaecology teams, guided ongoing care. At the time of last review, she remained oedematous but haemodynamically stable, with improving oxygenation on low-flow oxygen, and was scheduled for outpatient respiratory assessment and follow-up after her second rituximab infusion.

## Discussion

This case illustrates the diagnostic and therapeutic challenges posed by discordant serological and histopathological findings in nephrotic syndrome. Anti-PLA2R antibodies are present in approximately 70%-80% of patients with primary membranous nephropathy, and have high specificity for the disease [1,2]. In such cases, serology alone may suffice for diagnosis. However, our patient’s initial biopsy showed features of a resolving post-infectious glomerulonephritis, despite strongly positive PLA2R antibodies, creating uncertainty about the underlying pathology and delaying the start of immunosuppression.

Repeat renal biopsy four weeks later revealed classical features of membranous nephropathy, aligning histology with serology, and allowing targeted treatment with corticosteroids and rituximab. This sequence underscores the importance of reconsidering tissue diagnosis in cases where clinical, serological, and histological findings diverge. Mixed or evolving glomerular pathologies have been reported in elderly patients and may explain such discrepancies [3].

Management decisions were further complicated by the patient’s multisystem comorbidities. Her steroid-refractory microscopic colitis and rapidly progressive vitiligo reflected an underlying autoimmune milieu, but also increased the risk of immunosuppressive therapy. Severe diuretic-resistant oedema and recurrent nocturnal desaturation, due to previously undiagnosed COPD, limited her cardiorespiratory reserve and delayed procedures, including the repeat biopsy. Multidisciplinary coordination between nephrology, gastroenterology, dermatology, respiratory medicine, haematology, and gynaecology was therefore essential to optimise timing of interventions and balance risks versus benefits.

Previous reports have highlighted the diagnostic utility of PLA2R antibody titres for both disease activity and relapse prediction, but this case demonstrates that serology does not completely replace renal biopsy in complex presentations [4]. Guidelines recommend integrating serological, histological, and clinical data before initiating immunosuppression [5]. The efficacy and safety of rituximab in membranous nephropathy have been demonstrated in randomised trials [6].

Our patient’s good tolerance of prednisolone and rituximab, despite significant comorbidities, supports the feasibility of immunosuppressive therapy when the diagnosis is clarified and monitoring is close. This case adds to the limited literature on PLA2R-positive nephrotic syndrome with initially discordant histology, and reinforces the need for repeat biopsy when diagnostic doubt persists [7-9].

## Conclusions

In this patient, persistently elevated anti-PLA2R antibodies, with nephrotic-range proteinuria, initially contrasted with a renal biopsy suggesting post-infectious glomerulonephritis. Only by pursuing a second biopsy were we able to confirm membranous nephropathy and initiate disease-specific therapy with prednisolone and rituximab. Her course was further complicated by steroid-refractory microscopic colitis, progressive vitiligo, severe diuretic-resistant oedema, and new hypoxic episodes related to undiagnosed COPD, all of which delayed investigation and limited treatment options. Despite these challenges, a coordinated multidisciplinary approach allowed diagnosis to be clarified and immunosuppression to be commenced safely. This case emphasises how repeat tissue evaluation and close collaboration across specialties can directly alter management and improve outcomes in complex presentations of nephrotic syndrome.
